# Editorial: Integrating machine learning and AI in biological research: unraveling complexities and driving advancements

**DOI:** 10.3389/fbinf.2026.1806001

**Published:** 2026-03-10

**Authors:** Bindu Nanduri, Inimary Toby-Ogundeji

**Affiliations:** 1 College of Veterinary Medicine, Mississippi State University, Starkville, MS, United States; 2 Biology Department, Constantin College of Liberal Arts, University of Dallas, Irving, TX, United States

**Keywords:** artificial intelligence, biological, machine learning, omics, sequence analysis

## Introduction

The integration of Machine Learning (ML) and Artificial Intelligence (AI) is rapidly transforming biological research, providing sophisticated tools to analyze complex data, enhance precision, and navigate ethical considerations. This editorial summarizes five critical areas where AI is driving advancement, from foundational ethical shifts to deep prognostic insights in oncology.

Sandhu et al. discussed the foundational role of AI in ethical biomedical research. AI’s role transcends mere computational efficiency; it is a chief facilitator in ensuring humane and efficacious science by adhering to the “3Rs”: Replacement, Reduction, and Refinement, of animal-based research. This paper describes how traditional animal models have inherent limitations, including translational gaps, regulatory issues, and ethical controversies. AI provides the sophisticated analytical power necessary for predictions, simulations, and validations, minimizing reliance on animal subjects. By processing massive, complex datasets, machine and deep learning algorithms can simulate human biology, forecast therapy outcomes, and discover candidate drugs, thereby supporting Replacement and promoting Reduction through maximized experimental designs. This transition, however, necessitates strict validation requirements and ethical controls to ensure the reliability and integrity of the resulting models (Sandhu et al.).

Vázquez-González et al. focused their research work at driving precision diagnostics in Polymicrobial Diseases. One immediate challenge in biomedicine is the accurate classification of polymicrobial diseases caused by microbial community imbalance (dysbiosis), where 16S rRNA gene sequence data is highly dimensional and heterogeneous. To address this, the curated pipeline EPheClass was developed, utilizing ensemble-based ML models (including k-nearest neighbours (kNN), Random Forest (RF), Support Vector Machines (SVM), Extreme Gradient Boosting (XGBoost), and Multilayer Perceptron (MLP)) for binary phenotype classification. The methodology described in this article emphasizes rigorous procedures for reliability and reproducibility, unlike earlier studies criticized for insufficient sample size or lack of proper validation. Key data processing steps include Centred Log-Ratio transformation (CLR) for compositional data and Recursive Feature Elimination (RFE) for feature selection. This approach prioritizes model parsimony, demonstrating high predictive performance with a dramatically reduced number of features. For instance, using the Dynamic Ensemble Selection-Performance (DES-P) technique, EPheClass achieved an impressive Area Under the Curve (AUC) of 0.973 for diagnosing periodontal disease (PD) in saliva samples using just 13 features. The pipeline’s versatility was confirmed by successfully diagnosing Inflammatory Bowel Disease (IBD) (using 26 features) and classifying antibiotic exposure (DA) (using 22 features), demonstrating its generalization across different phenotypes and sample types (Vázquez-González et al.).

The goal of this research was to unravel cross-omics interactions, specifically Predicting miRNA from mRNA. The authors addressed a gap within the lack of publicly available paired datasets containing both miRNA and mRNA expression profiles. The authors’ evaluation process consisted of seven paired datasets related to viral infections, specifically West Nile Virus (WNV) and Human Immunodeficiency Virus (HIV). Overall, both DNNs and LASSO models achieved strong correlations at the level of individual samples. However, DNNs proved superior in capturing predictive changes relevant to differential expression analysis (DEA). Specifically, cross-study validation using HIV datasets yielded strong correlations for log-fold changes (log2FCs) derived from DEA (R = 0.59), demonstrating the model’s ability to generalize to independent data of the same tissue type. Furthermore, data augmentation, specifically adding Gaussian noise, consistently improved the performance of the neural networks, helping mitigate the challenge of small sample sizes. Conversely, linear LASSO models, despite their strong sample-level performance, struggled to translate this accuracy into meaningful correlations for DEA log2FCs, suggesting the non-linear capability of DNNs is better suited for complex cross-omics relationships (Böge et al.).

The authors presented a powerful computational framework for Lung Adenocarcinoma (LUAD) Prognosis. This framework integrated multi-omics data (transcriptomic, DNA methylation, and somatic mutation data) with 10 clustering algorithms to identify three robust molecular subtypes (CS1, CS2, and CS3) associated with distinct clinical prognoses (CS3 having the best prognosis). Leveraging 10 ML algorithms in 101 unique combinations, researchers constructed the PIGRS (Lasso + GBM ensemble) prognostic model based on 15 immune-associated programmed cell death genes (PIRGs). PIGRS demonstrated strong prognostic efficacy across multiple cohorts, outperforming almost all previously published LUAD prognostic models. The model linked high PIGRS scores to increased genomic instability, including higher Tumor Mutational Burden (TMB) and intra-tumor heterogeneity (MATH scores), and suggested a relationship with immune escape. Subsequent experimental validation showed that knockdown of PSME3, significantly inhibited LUAD cell proliferation, migration, and invasion, and promoted apoptosis likely by affecting the PI3K/AKT/Bcl-2 signaling pathway (Xie et al.).

The authors focused on innate immune cell barrier-related genes to inform prognosis for pancreatic cancer (PC). Using 14 machine learning algorithms, the CDRG-RSF model (Random Survival Forest trained on risk genes) was established as the most robust prognostic tool, achieving excellent long-term predictive performance with 3-year and 5-year AUCs exceeding 0.7 in validation cohorts. High-risk PC patients exhibited elevated TMB and reduced infiltration of anti-tumor cytotoxic cells, specifically NK and CD8^+^ T cells. The model offered actionable therapeutic insights: high-risk patients showed resistance to Erlotinib and Oxaliplatin but increased sensitivity to 5-Fluorouracil. Five key prognostic genes were identified, including UBASH3B, a novel marker that exhibited a significant negative correlation with NK cell activation and appeared to mediate immune signaling and drug resistance, positioning it as a potential target for personalized therapy (Lou et al.).

## Conclusion

Taken together, the convergence of ML/AI/biological research provides scientists with the algorithmic lenses necessary to filter complex, high-dimensional biological data into clinically actionable knowledge, moving the field rapidly toward precision medicine. These advancements promise a future where precision medicine, agricultural approaches, environmental impacts, etc., are informed by highly validated, robust, and reproducible computational frameworks, pushing the boundaries of discovery while upholding the highest standards of scientific ethics and rigor. This transformative collaboration is not just an incremental step but a fundamental leap towards solving the most challenging biological puzzles.

